# The complete mitochondrial genome of *Lumbricus rubellus* (Oligochaeta, Lumbricidae) and its phylogenetic analysis

**DOI:** 10.1080/23802359.2019.1644242

**Published:** 2019-07-22

**Authors:** Qingzheng Zhang, Hongyi Liu, Yufeng Zhang, Honghua Ruan

**Affiliations:** a College of Biology and the Environment, Nanjing Forestry University, Nanjing, China;; b Hebei Key Laboratory of Animal Diversity, Langfang Normal University, Langfang, China

**Keywords:** *Lumbricus rubellus*, mitochondrial genome, next generation sequencing, phylogenetic analysis

## Abstract

The complete mitochondrial genome of *Lumbricus rubellus* was analyzed by next-generation sequencing. The mitogenome was 15,464 bp in length, comprising 13 protein-coding genes (PCGs), 22 transfer RNAs, 2 ribosomal RNAs, and a non-coding region. The phylogenetic analysis of 13 PCGs within the class Oligochaeta suggested that *L. rubellus* was placed as sister to *L. terrestris* of the same genus. The results obtained here can contribute to the phylogenetic analysis of earthworms.

*Lumbricus rubellus* belongs to the Lumbricidae family of the Oligochaeta subclass. *Lumbricus rubellus* is native to Europe and is also common in the Northern Palearctic region, including the far east of Russia. It has now been introduced into other regions worldwide, such as North America, Australia, and New Zealand (Shepeleva et al. 2008). Like other earthworms, *L. rubellus* is ecologically and environmentally important. (Eisenhauer and Scheu 2008; Oldenburg et al. 2008; Laossi et al. 2010). As a model organism, it is extensively employed to investigate toxic mechanisms and environmental modification (Guo et al. 2009). For this study, the complete mitochondrial genome of *L. rubellus* was assembled and characterized, which could provide a better understanding of the genetics and evolutionary process of *L. rubellus* and other earthworm species.

*Lumbricus rubellus* samples were obtained from a suburb of Khabarovsk, Russia (48°29′N, 135°05′E). Following morphological identification, the specimens were stored at −20 °C in the Zoology Laboratory of Nanjing Forestry University (Accession: HB20170803). Total genomic DNA was extracted using Insect DNA Kit (Omega Bio-tek, Norcross, GA, USA). The genomic library was established using an Illumina TruSeq™ Nano DNA Sample Prep Kit (Illumina, San Diego, CA, USA) and sequenced using the Illumina Hiseq2000 (Illumina, USA). The low-quality sequences were removed to generate clean data, which had 44,664,406 reads and a GC content of 41.7%. The assembled mitogenome was obtained using ABySS v2.0.2 and GapCloser v1.12 (Nagarajan and Pop 2013) whereas partial segments were examined via Sanger sequencing. The complete mitochondrial genome of *L. rubellus* was 15,464 in size and its overall base composition was 31.6% for A, 32.1% for T, 21.8% for C, and 14.5% for G, with a GC content of 36.3%. This included 13 unique protein-coding genes (PCGs), 22 transfer RNA genes, 2 ribosomal RNA genes, and a non-coding region. The total length of the PCGs was 10,940 and the average length was 842 with a GC content of 37.5%. All of the details were listed in GeneBank (Accession: MN102127) and most of the PCGs began with a common Met start codon, while ND1 and ND4L were encoded with Ile.

The phylogenetic relationship of *L. rubellus* among the class Oligochaeta was determined from a concatenated dataset, including the 13 PCGs, using Mrbayes v3.2.5 software (Huelsenbeck et al. 2002). The phylogenetic tree revealed that *L. rubellus* was clustered most intimately with *L. terrestris* of the same genus (Figure 1). The mitogenome obtained here will assist with elucidating the genetic diversity, evolutionary origin, and genetic relationships of earthworms.

**Figure 1.**
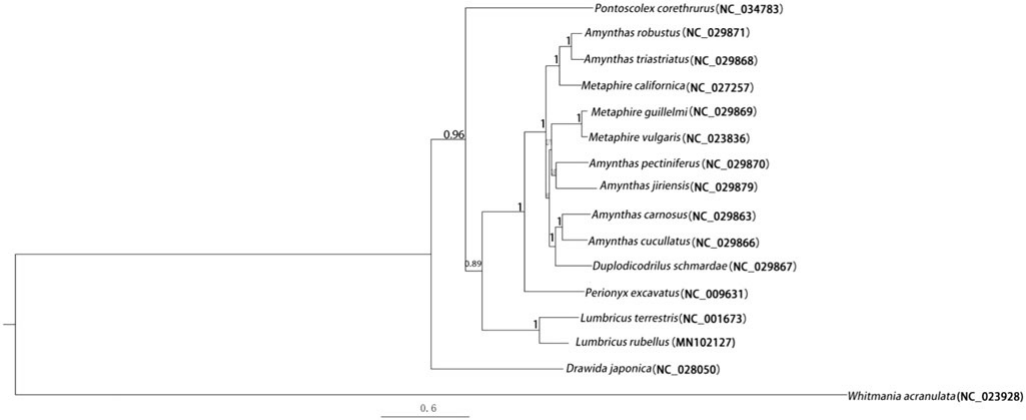
Phylogenetic tree based on the 13 PCGs of 16 species of Oligochaeta inferred by the Bayesian method. Posterior probability values are shown on the nodes.
